# Supplementary material for: Long Non-Coding RNA-Based Functional Prediction Reveals Novel Targets in Notch-Upregulated Ovarian Cancer
Source: Cancers (Basel). 2022 Mar 18;14(6):1557. doi: 10.3390/cancers14061557 (PMC8946805; doi:10.3390/cancers14061557)
Supplement: Supplementary file 1 [file cancers-14-01557-s001.zip › cancers-1628282-supplementary.pdf]

cancers

Supplementary Materials

Long Non-coding RNA-Based Functional Prediction Reveals Novel Targets in Notch-Upregulated Ovarian Cancer

Seonhyang Jeong[1,†], Sunmi Park[1,†], Young Suk Jo[1], Moon Jung Choi[2], Gibbeum Lee[2], Seul Gi Lee[3], Min Chul Choi[4], Hyun Park[4], Won Duk Joo[4], Sang Geun Jung[4*] and Jandee Lee[2*]

1 Department of Internal Medicine, Yonsei University College of Medicine, 50-1 Yonsei-ro, Seodaemun-gu, Seoul 03722, South Korea BAMBI_89@yuhs.ac (S.J.); SUNMIP@yuhs.ac (S.P.); joys@yuhs.ac (Y.-S.J.)
2 Department of Surgery, Open NBI Convergence Technology Research Laboratory, Severance Hospital, Yonsei Cancer Center, Yonsei University College of Medicine, 50-1 Yonsei-ro, Seodaemun-gu, Seoul 03722, South Korea; dnflc2000@naver.com (M.-J.C); GBLEE@yuhs.ac (G.L)
3 Department of Surgery, Eulji University School of Medicine, 95 Dunsanseo-ro, Seo-gu, Daejeon 35233, South Korea, rtigger@naver.com (S.-G.L.)
4 Department of Gynecological Oncology, Comprehensive Gynecologic Cancer Center, CHA University, 59, Yatap-ro, Bundang-gu, Seongnam Gyeonggi-do 13496, South Korea; oursk79@cha.ac.kr (M.-C.C); p06162006@cha.ac.kr (H.P.); wdjoo@cha.ac.kr (W.-D.J.)
* Correspondence: JL: JANDEE@yuhs.ac; SGJ: sgoncol@chamc.co.kr
† These two authors contributed equally to this work.

This file includes Table S1 and Figures S1-S5.

Table S1. Primer sequences used for the RT-PCR.

Gene Name		Primer Sequence
GAPDH	Forward	GCCGTCTAGAAAAACCTGCC
	Reverse	ACCACCTGGTGCTCAGTGTA
TAF1C	Forward	GCCTGAAAGCACCGACCATA
	Reverse	GTAGAAGACATCTCCCGCCG
ADCY6	Forward	CACATAGCACCGCAGTTGGCAT
	Reverse	AGGCAGTGATGTCAGCAGGTGT
DGCR8	Forward	CAAGCAGGAGACATCGGACAAG
	Reverse	CACAATGGACATCTTGGGCTTC
RAD52	Forward	ATAAGTAGCCGCATGGCTGG
	Reverse	ATCCACATTCTGCTGCGTGA
ACRV1	Forward	GCATGGTTCAAGCAAGCACA
	Reverse	CCTTCAGCATGTTCAGTCGC
NPY5R	Forward	GCTGGATCAGTGGATGTTTGG
	Reverse	CAGATGGCAAAACCTAGTGTCC
DMC1	Forward	CCGGGAGCCAGGAATTTGAT
	Reverse	TTGAGCTGTCACACAGAGGG
TCP11	Forward	CTCACCATGTCACCTCCGAC
	Reverse	CAAGAAGCCCTGACACAGCA
NOTCH1	Forward	GACAGCCTCAACGGGTACAA
	Reverse	GCCACTGGTCATGTCTTTGC
NOTCH3	Forward	CCAACCTGGCAGGGAGTTTC
	Reverse	CACCGTTCAGGCATGGGTTG
HES1	Forward	AAAAATTCCTCGTCCCCGGT
	Reverse	ATGCCGCGAGCTATCTTTCT
HEY1	Forward	TCGGCTCTAGGTTCCATGTC
	Reverse	GCTTAGCAGATCCCTGCTTCT
LINC01572	Forward	GGAAGGACACCATTGACAGC
	Reverse	GGTTGGTGCCTGACTTTTGT
GUSBP11	Forward	CTGCTAGAGCAGTACCATCTGG
	Reverse	GTAGTCTTGGGCTGACACTGGT
AC069277.1	Forward	TGTGCTCAAGGTGGTCAGAG
	Reverse	AGTTGTCTCGCCTTTCCAGA
ERVK13-1	Forward	CCAGGATGCGACTTTTGTTT
	Reverse	TACTCGGGTGGTTGAGGAAG
AC087294.1	Forward	AAAGTTAGCTGGGCATGGTG
	Reverse	TGATCTTGGCTCACTGCAAC

Abbreviations: GAPDH, glyceraldehyde-3-phosphate dehydrogenase; TAF1C, TATA-box binding protein associated factor, RNA polymerase I subunit C; ADCY6, Adenylate Cyclase 6; DGCR8, DGCR8 Microprocessor Complex Subunit; RAD52, RAD52 Homolog, DNA Repair Protein; ACRV1, Acrosomal Vesicle Protein 1; NYP5R, Neuropeptide Y Receptor Y5; DMC1, DNA Meiotic Recombinase

1; TCP11, T-Complex 11; NOTCH1, Notch Receptor 1; NOTCH3, Notch Receptor 3; HES1, hes family bHLH transcription factor 1; HEY1, Hairy/enhancer-of-split related with YRPW motif protein 1; LINC01572, Long Intergenic Non-Protein Coding RNA 1572; GUSBP11, GUSB Pseudogene 11; AC069277.1, ENSG00000189229 Gene lncRNA; ERVK13-1, Endogenous Retrovirus Group K13 Member 1; AC087294.1, novel transcript, antisense to TMEM11.

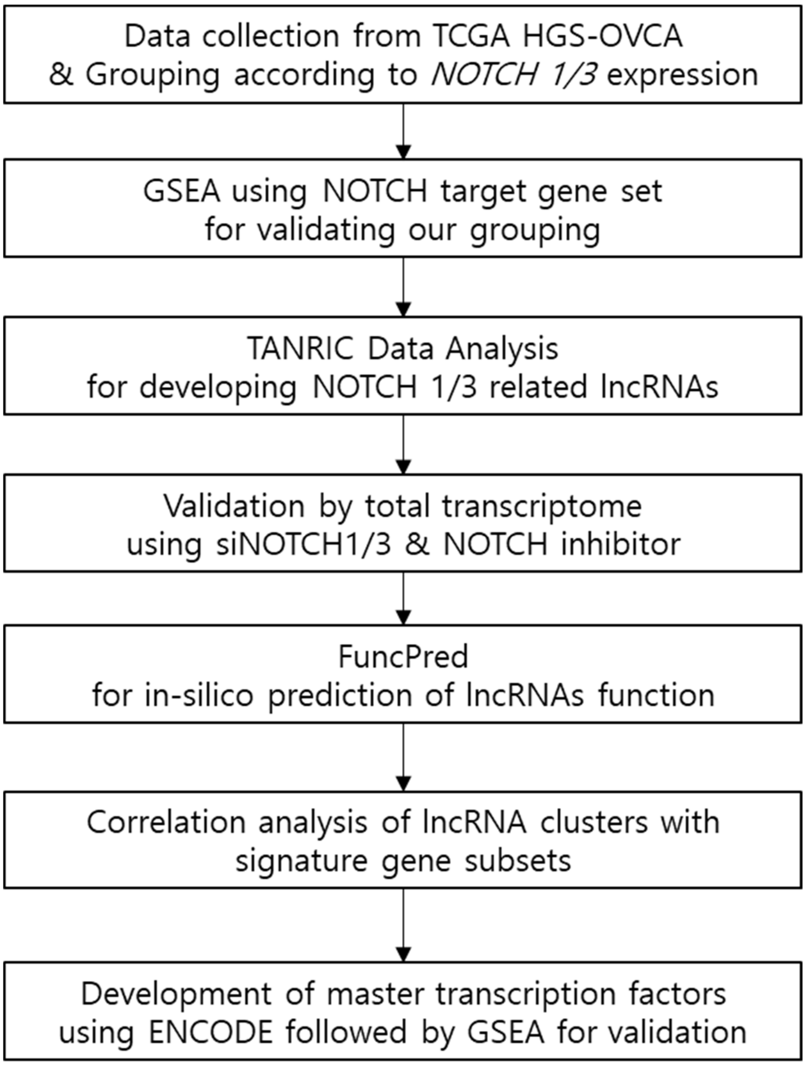

Figure S1. Scheme of our analytic strategy.

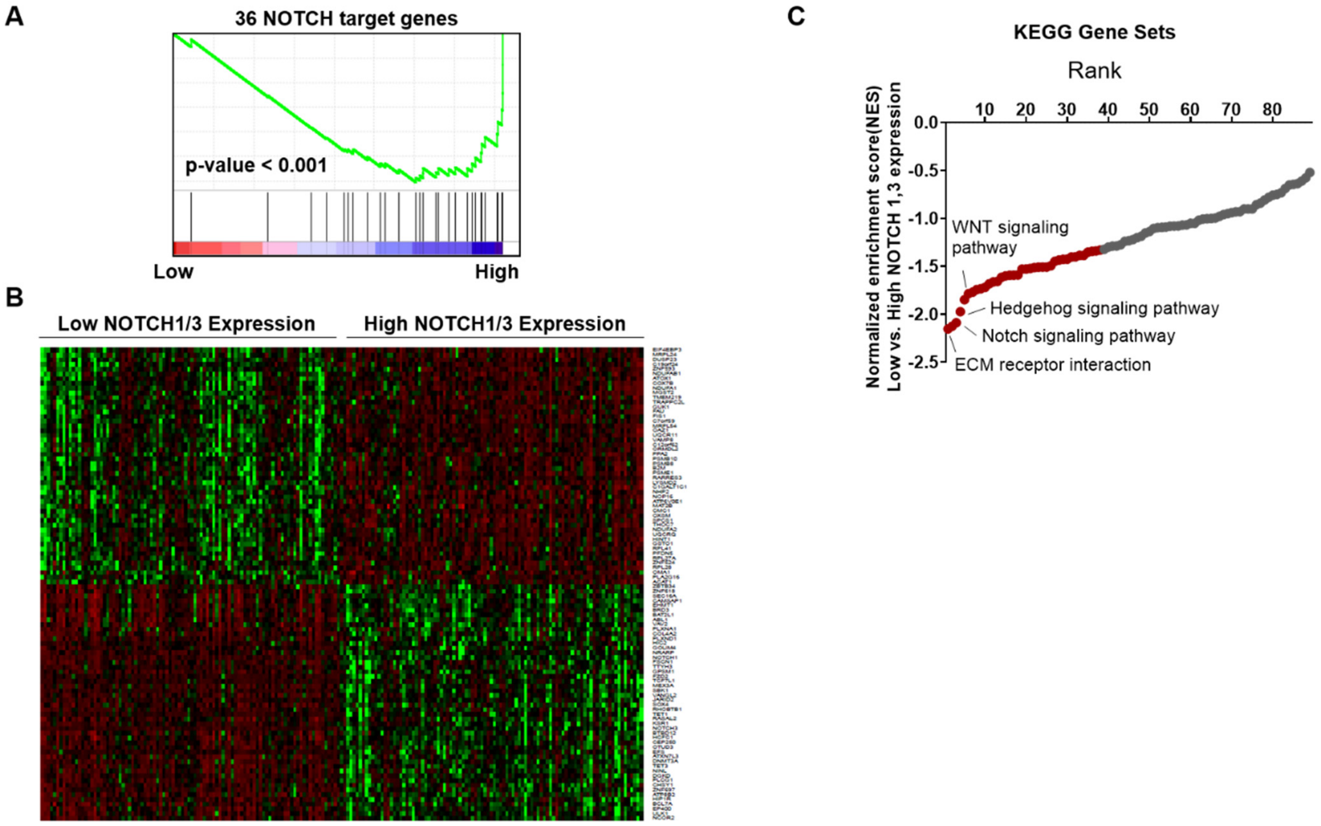

Figure S2. Grouping of ovarian cancer according to *NOTCH1/3* mRNA expression status derived from TCGA HGS-OVCA (N=303). (A) GSEA results using Notch target gene set (gene number = 36) indicating highly enriched in NOTCH1/3 upregulated ovarian cancer. (B) Heatmap using representative genes showing highly differential expression (n=100) in high and low NOTCH1/3 mRNA expression groups. (C) GSEA results indicating highly enriched gene sets in NOTCH1/3 upregulated ovarian cancer using KEGG gene sets. “High” indicates a group with high expression of both *NOTCH1* and *NOTCH3* (n=97). “Low” indicates a group with low expression of both *NOTCH1* and *NOTCH3* (n=96). The criteria for dividing high and low were median TPM values. High NOTCH1 with low NOTCH3 (n=55) and high NOTCH3 with low NOTCH1 (n=55) were excluded. Abbreviations: NOTCH1/3, Notch receptor 1 and 3; TCGA HGS-OVCA, The Cancer Genome Atlas (TCGA) high grade serous ovarian cancer; GSEA, gene set enrichment analysis; KEGG, Kyoto Encyclopedia of Genes and Genomes.

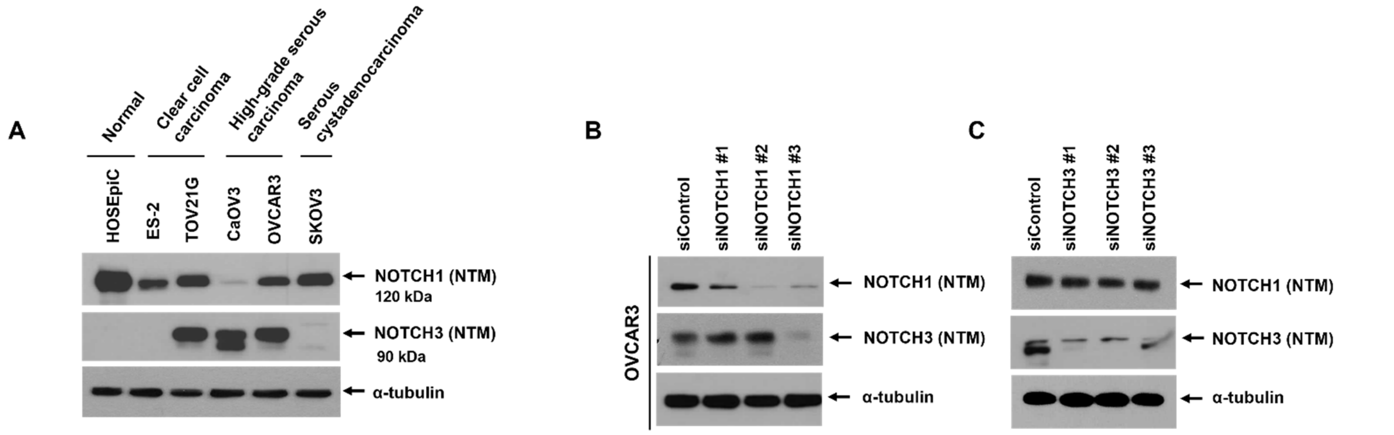

Figure S3. Validation of the silencing effect of si*NOTCH1* and si*NOTCH3*. (A) Expression of NOTCH1 and NOTCH3 in normal ovarian surface epithelial cell line (HOSEpic cells), clear cell carcinoma cell line (ES-2 and TOV21G cells), high-grade serous carcinoma (Caov3, OVCAR3), and serous cystadenocarcinoma (SKOV3 cells). Representative western blot analysis showing knock-down effect of siNOTCH1 (B), siNOTCH3 (C) in OVCAR3 cell line.

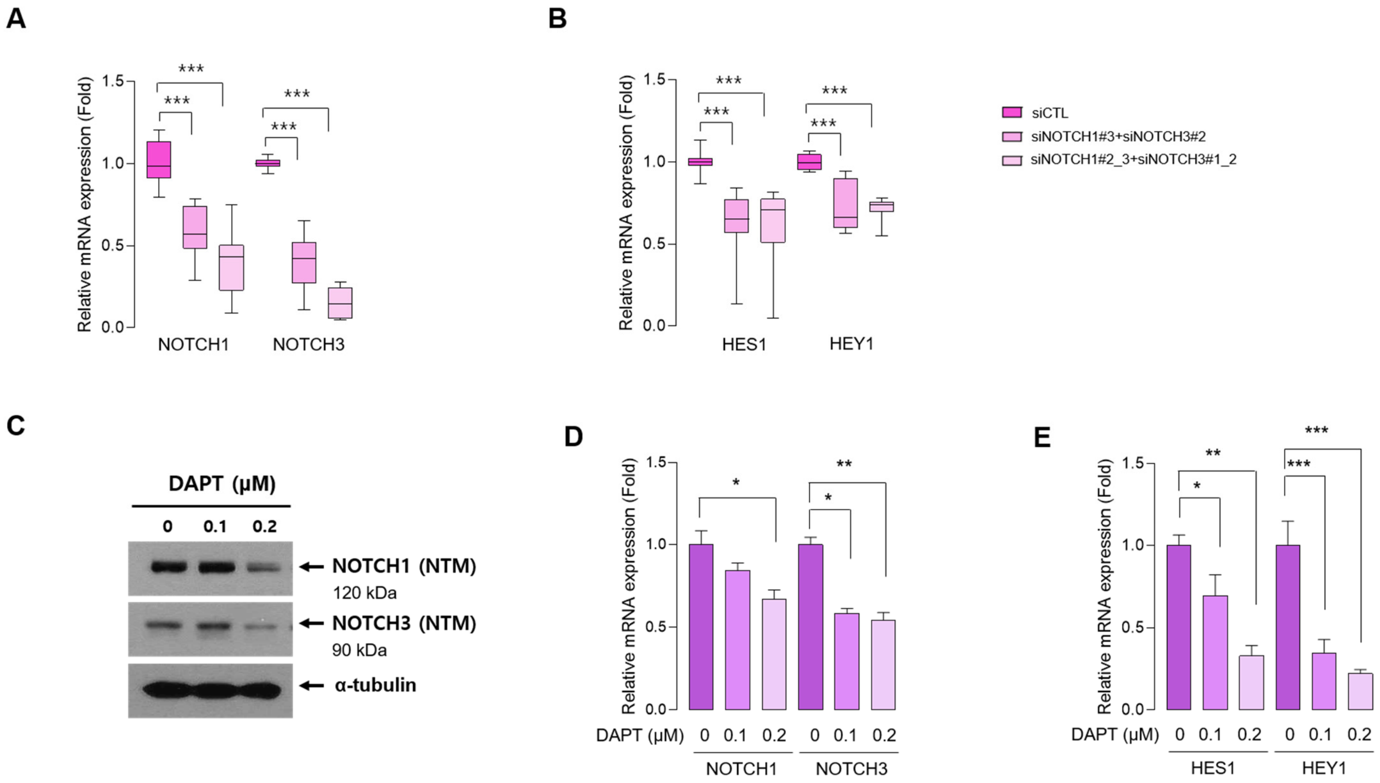

Figure S4. NOTCH1 and NOTCH3 knock-down by siRNA and NOTCH inhibitor treatment (DAPT). **A-B.** qRT-PCR for *NOTCH1/3* (A), *HES/HEY1* (B) in OVCAR3 cells transfected with control siRNA (siCTL) or NOTCH 1/3 siRNAs (siNOTCH1/3). **C-E.** Results from western blot analysis for NOTCH1 and NOTCH3 (NTM) (C) and qRT-PCR for *NOTCH1/3* (D), *HES/HEY1* (E) in OVCAR3 cells treated by indicated concentration of DAPT for 48hr. Data was shown as mean ± SD and p-values were calculated by two-tailed Mann-Whitney U-test. All experiments were repeated three times, and each experiment was performed in triplicates. **P < 0.05, **P < 0.01, ***P < 0.001.* Abbreviations: NTM, transmembrane/intracellular region; DAPT, N-[N-(3,5-difluorophenacetyl)-L-alanyl]-S-phenylglycine t-butyl ester.

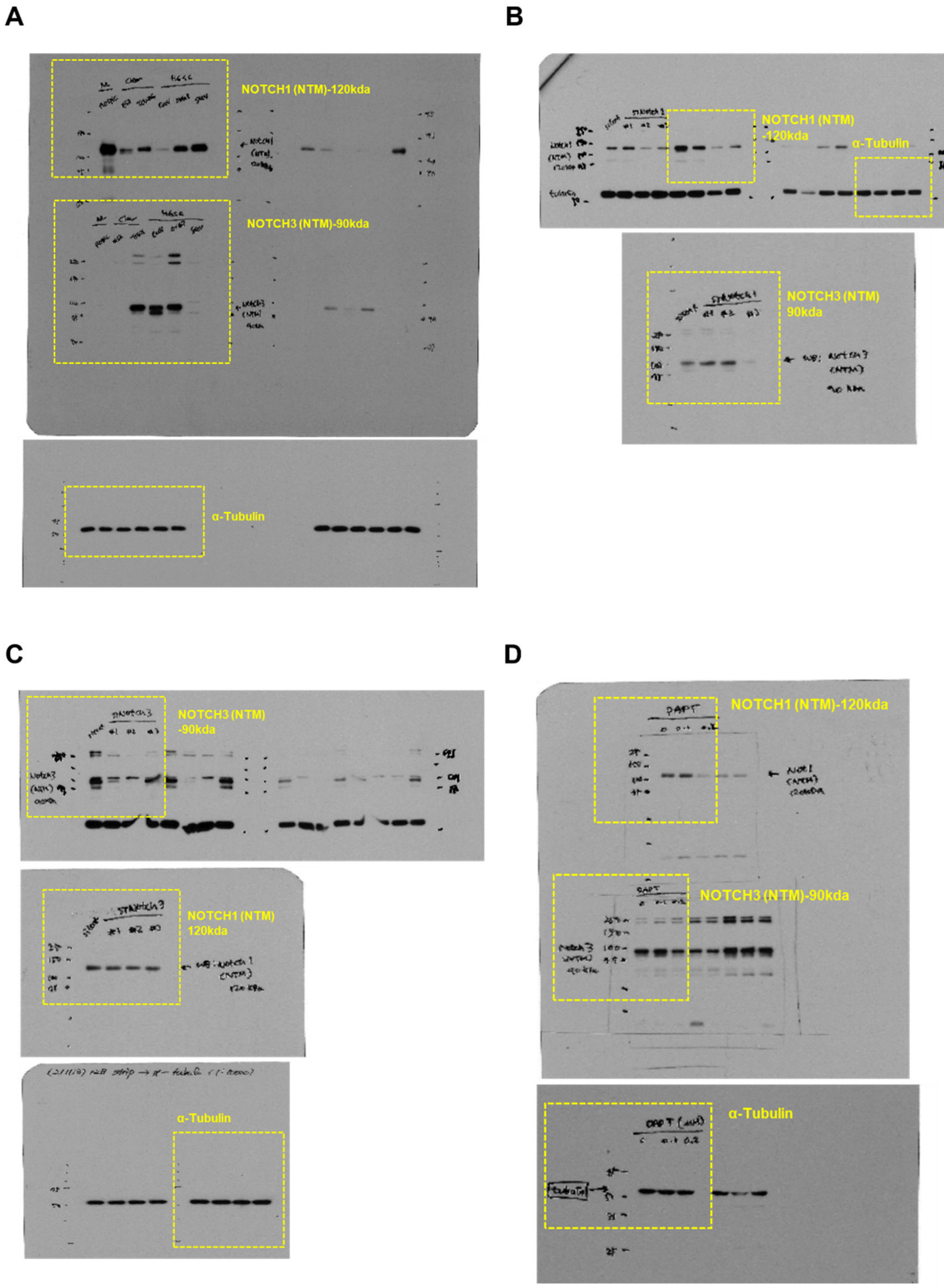

Figure S5. Original uncropped western blot images related to Figures. (A) Original uncropped western blot images related to Figure.S3A, (B) Figure. S3B, (C) Figure. S3C. (D) Original uncropped western blot images related to Figure. S4C.
